# Contactless exercise intervention in prenatal and postnatal period during COVID-19 lowers the risk of postpartum depression

**DOI:** 10.1038/s41598-024-60658-7

**Published:** 2024-04-29

**Authors:** Dong-Joo Hwang, Joon-Yong Cho, Ah-Hyun Hyun

**Affiliations:** 1 Exercise Biochemistry Laboratory, Korea National Sport University, Seoul, Korea; 2 Sport Science Institute, Korea National Sport University, Seoul, Korea

**Keywords:** Health care, Risk factors

## Abstract

The COVID-19 pandemic has had a substantial adverse impact on the physical and mental health of pregnant and postpartum women, thereby increasing the risk of postpartum depression (PPD). This study aimed to evaluate the effectiveness of a continuous contactless exercise intervention in reducing the risk of depression during the prenatal and postnatal periods. The study utilized an interactive contactless exercise program consisting of Pilates movement over a 16-week period, with 8 weeks during pregnancy and 8 weeks after childbirth. Metabolic and psychological factors related to postpartum depression, including pain, stress, and stress-response markers, were analyzed. The results showed that the exercise intervention significantly alleviated postpartum depression by improving pain (Oswestry Disability Index: Non-exercise, 11.4 ± 14.8 versus Exercise, − 63.1 ± 18.4, *p* < .001) and stress factors (Edinburgh Postnatal Depression Scale: Non-exercise, 8.8 ± 8.72 versus Exercise, − 37.6 ± 9.13, *p* < .001; Perceived Stress Scale: Non-exercise, 9.21 ± 9.35 versus Exercise, − 20.7 ± 14.4, *p* < .001) caused by physical/structural imbalances in postpartum women. Additionally, the intervention improved the metabolic imbalances commonly observed after childbirth, including reductions in triglyceride (Interaction effect, *p* = .017), insulin (Interaction effect, *p* = .032), and cortisol levels (Interaction effect, *p* < .001), which are recognized risk factors for postpartum depression. Taken together, these findings suggest that contactless online exercise interventions can mitigate postpartum depression by addressing metabolic dysregulation that frequently occurs after delivery, especially in situations of social isolation caused by the pandemic.

## Introduction

The coronavirus (COVID-19) pandemic has brought many changes to our daily lives, culture, society, and economy worldwide^1^. As a precautionary measure against infection, most people chose to stay at home, leading to notable rise in obesity and related complications due to reduced physical activity^2^. In particular, pregnant women, considered as high-risk group with unstable psychological conditions due to changes in hormones, were excluded from vaccination, thereby increasing psychological burdens such as fear of infection and anxiety^3,4^; quarantine also increased the level of physical inactivity and the risk of postpartum depression^5,6^. To prevent these problems, maternal care, such as prenatal care, meditation, and exercise, was implemented; however, as all educational programs were suspended after pandemic, women's health was widely threatened^7^.

Postpartum depression is a mental illness that has negative consequences on the mother's health status and the physical development of offspring after childbirth; approximately 40% of women experience postpartum depression after childbirth^8^. The pathological mechanisms of postpartum depression have not been clearly identified; however, physical problems such as repetitive housework, back pain, obesity, lack of sleep, loss of self-identity, appearance dissatisfaction, and environmental factors can cause postpartum depression^9,10^. In relation to COVID-19, increased stress during the pandemic had a substantial impact on postpartum depression outbreaks, and the resurgence of the virus further increased stress, anxiety, and depression levels in women^11,12^.

As such, the development of postpartum depression is influenced by a combination of various causes, and physical activity is recommended for both preventive and therapeutic purposes^13,14^. In pregnancy, women undergo a shift in the center of mass due to fetal growth and weight gain, resulting in increased stress on the lower back. Furthermore, pregnancy-related hormones, such as relaxin, relax the pelvic ligaments, diminishing pelvic stability^15^. Therefore, exercise for pregnant women should prioritize minimizing pain associated with biomechanical changes and adopting safe activities. Numerous studies have established the benefits of exercise, and Pilates, known for a combined form of aerobic and anaerobic movements, emerges as a particularly effective practice for enhancing women’s health both before and after childbirth, with references providing support for its effectiveness in addressing various aspects^16,17^. Studies emphasizes its ability to correct body balance by strengthening core and pelvic muscle around the hip joints and alleviate back pain in pregnant women^18,19^. Consistently, participating in Pilates contributes to reducing levels of triglycerides (TG), low-density lipoprotein (LDL), cholesterol, and insulin resistance, thus acting as a preventive measure against gestational obesity. It has also exhibited efficacy in alleviating apprehensions related to childbirth, anxiety, fatigue, and stress during pregnancy^20^. Furthermore, the benefits of Pilates extend into the postpartum period, with a reported significant effect on weight control and reduction of waist-hip ratio (WHR) after childbirth, and its concomitant reduction in visual analog scale (VAS) and Oswestry Disability Index (ODI) score, indicator related to back pain^21,22^.

However, after COVID-19, it has become difficult for women to exercise, and there is a significant lack of alternative methods for prenatal management^23^. In accordance with the American College of Obstetrics and Gynecologists (ACOG) recommendation of a minimum of 150 min of exercise per week during pregnancy and postpartum period^24^, Silva-Jose et al. suggested that physical activity through virtual modalities could be a suitable alternative, especially in situations where physical activity may be restricted^25^. Several studies have verified the effect of non-face-to-face exercise^26–29^. Only a few studies are available on online exercise in pregnant women during COVID-19; Ramachandra found that tele-physical therapy in 29 weeks pregnant women with severe pelvic girdle pain reduced back pain, and Pilates, with the help of a real-time video chat application, reduced back pain and pelvic pain in women after childbirth^21,22,28^. In addition, postpartum online Pilates was effective in preventing PPD and diet effects^28^. These results suggest that prenatal and postpartum exercises are effective in preventing depression, regardless of the form (face-to-face or non-face-to-face). Nevertheless, women are not properly managed, and epidemiological investigations of postpartum depression over the past two to three years are insufficient^30^. Ebrahimian et al*.* reported that 90% of postpartum women experience a feeling of melancholy, which is a pre-depression stage, but most of them do not recognize it as a disease; missing treatment for severe postpartum depression can lead to serious social problems such as loss of judgment, infant murder, or suicide^8^. Accordingly, the guidelines from the American College of Obstetricians and Gynecologists (ACOG) emphasizes the necessity of monitoring women's mental health for one year after childbirth, specifically underscoring the need for periodic monitoring for postpartum depression^31,32^.

Considering that postpartum depression is caused by emotional problems in pregnant women and failure to manage prenatal conditions, including obesity, and that biochemical changes related to pregnancy obesity are closely correlated with postpartum psychological diseases^3^, continuous exercise participation from pregnancy to the postpartum period can act as an important variable. However, to date, most postpartum depression-related studies have been conducted separately in pregnant and postpartum women, and few studies have examined the relationship between postpartum depression and biological changes in pregnant women, reflecting environmental characteristics such as COVID-19. Therefore, this study aimed to investigate the effect of continuous online training before and after childbirth, on the risk factors of postpartum depression and to determine its correlation with obesity.

## Results

### Changes of body composition in non-exercised and exercised postpartum women.

Postpartum women typically experience changes in body composition, and these changes might have various health implications. Therefore, understanding the association between body composition and postpartum women is crucial for identifying the potential health risks and developing therapeutic options. The body composition parameters measured before and after the continuous online exercise program in postpartum women are presented in Table 1. No significant group-by-time interactions were observed in body composition parameters (Table 1). However, in the exercised group, beneficial changes in the fat ratio were observed in comparison with the non-exercised group. The effect size, as measured by Cohen’s d, was d = 0.86, indicating a larger effect. Unexpectedly, the exercise group showed a decrease in lean mass; however, the effect size was d = 0.23, indicating a small effect. Thus, our results suggest that continuous exercise participation using an online platform in a socially isolated situation increases the body’s metabolic rate, which helps postpartum women to burn more calories and reduce body fat.

**Table 1:** Comparison of body composition and metabolic parameters in blood before and after contactless exercise.

	Non-exercise		Effect size	Exercise		Effect size	Statistics		
	Pre	Post	Cohen's d	Pre	Post	Cohen's d	Interaction effect	Time effect	Group effect
Body weight (g)	65.43 ± 6.8	63.47 ± 6.8	0.40	66.84 ± 6.0	60.68 ± 7.1	1.32	F (1, 116) = 2.834; *p* = .095	F (1, 116) = 10.57; *p* = .002	F (1, 116) = 0.3011; *p* = .584
Fat mass (g)	25.05 ± 5.5	23.54 ± 5.7	0.38	23.93 ± 5.2	20.75 ± 5.6	0.83	F (1, 116) = 0.6572; *p* = .419	F (1, 116) = 5.212; *p* = .024	F (1, 116) = 3.623; *p* = .059
Lean mass (g)	24.75 ± 6.0	24.61 ± 5.6	0.03	29.14 ± 8.7	27.76 ± 8.1	0.23	F (1, 116) = 0.2138; *p* = .645	F (1, 116) = 0.3179; *p* = .574	F (1, 116) = 7.823; *p* = .006
Fat ratio (%)	36.41 ± 4.7	34.86 ± 4.7	0.46	34.55 ± 4.9	31.55 ± 4.9	0.86	F (1, 116) = 0.6610; *p* = .418	F (1, 116) = 6.411; *p* = .013	F (1, 116) = 8.300; *p* = .005
BMI (kg/m^2^)	25.59 ± 2.9	24.60 ± 3.1	0.46	25.49 ± 2.7	23.22 ± 3.3	1.04	F (1, 116) = 1.278; *p* = .261	F (1, 116) = 8.173; *p* = .005	F (1, 116) = 1.691; *p* = .196
TG (mg/dL)	210.1 ± 56.8	157.20 ± 49.1	1.41	199.30 ± 61.7	99.53 ± 36.3	2.79	F (1, 116) = 5.907; *p* = .017	F (1, 116) = 62.68; *p* < .001	F (1, 116) = 12.61; *p* < .001
TC (mg/dL)	236.70 ± 30.2	200.07 ± 28.7	1.75	241.63 ± 36.2	183.40 ± 33.9	2.35	F (1, 116) = 3.214; *p* = .076	F (1, 116) = 62.00; *p* < .001	F (1, 116) = 0.9485; *p* = .332
HDL (mg/dL)	71.63 ± 17.9	60.97 ± 16.6	0.87	76.00 ± 15.6	72.93 ± 19.1	0.25	F (1, 116) = 1.385; *p* = .242	F (1, 116) = 4.523; *p* = .036	F (1, 116) = 6.398; *p* = .013
LDL (mg/dL)	152.13 ± 25.4	126.13 ± 22.7	− 1.52	148.00 ± 25.0	111.23 ± 26.7	− 2.00	F (1, 116) = 1.338; *p* = .250	F (1, 116) = 45.47; *p* < .001	F (1, 116) = 4.181; *p* = .043
CRP (mg/dL)	1.02 ± 0.7	0.68 ± 0.6	− 0.67	0.87 ± 0.8	0.26 ± 0.3	− 1.37	F (1, 116) = 1.124; *p* = .291	F (1, 116) = 14.32; *p* < .001	F (1, 116) = 5.256; *p* = .024

Two-way ANOVA followed by Tukey’s multiple comparison test was performed. Tables depict mean ± SD. Pairwise calculation of (Cohen's d) effect size between pre- and post-values were expressed as mean changes and its values are considered as small (0.2), medium (0.5), or large (0.8 or greater) effective size. *p* < 0.05 was considered to be statistically significant.

### Impact of continuous home-based contactless exercise participation via an online platform on metabolic parameters in postpartum women

During pregnancy, a women’s body undergoes significant changes in lipid metabolism, which may persist for some time after delivery. Continuous exercise participation via an online platform altered the metabolic parameters, showing a tendency to decrease in both the non-exercised and exercised groups of postpartum women (Table 1). A significant group-by-time interaction was identified only for TG level (*F*_(1,113)_ = 5.907, *p* = 0.017, two-way ANOVA followed by Bonferroni’s post hoc test). During the postpartum period, all participants exhibited a significant decrease in TG levels (*F*_(1,113)_ = 62.68, *p* < 0.001, time effect in two-way ANOVA followed by Bonferroni’s post hoc test), with a large effect size (d = 2.79); however, only postpartum women who exercised, showed a greater reduction in TG levels compared to the non-exercised group (*F*_(1,113)_ = 12.61, *p* < 0.001, group effect in two-way ANOVA followed by Bonferroni’s post hoc test). In terms of high-density lipoprotein (HDL), it is generally expected that exercise is effective in increasing the level of HDL; however, the results showed a decreasing tendency in both the non-exercise and exercise group, although that of the exercise group was significantly higher (*p* = 0.048, Bonferroni’s multiple comparison test, Table 1).

### Hormonal changes mediated by continuous contactless exercise via an online platform during postpartum period

**Figure 1:**
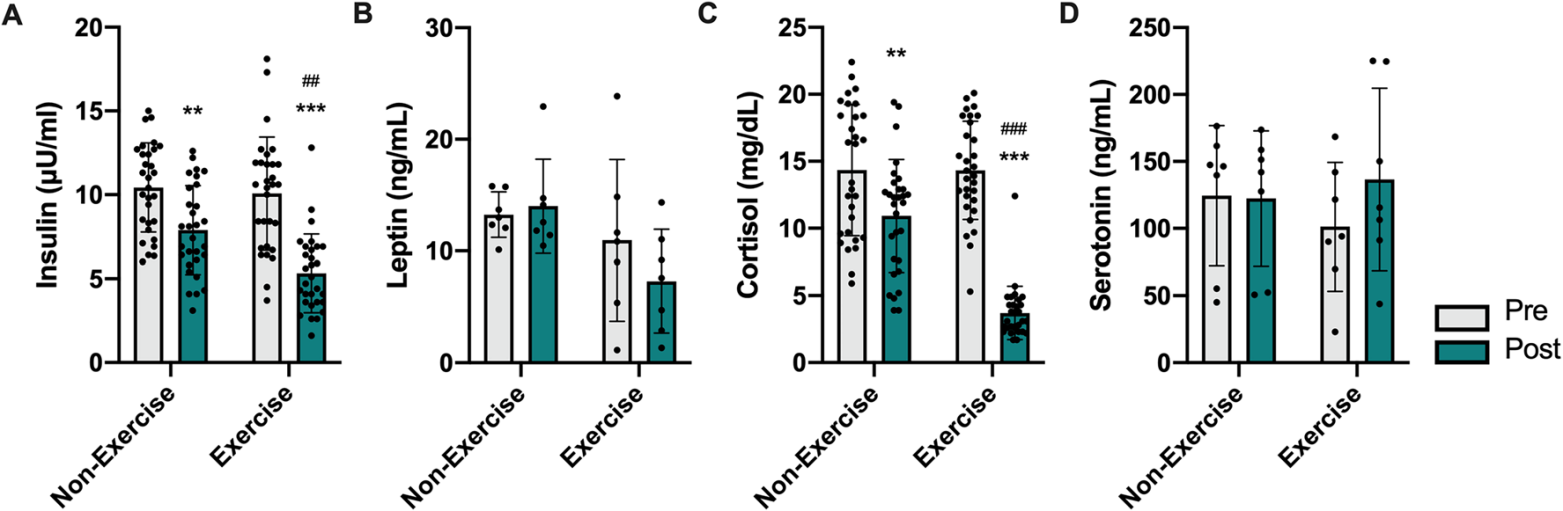
Effect of the continuous home-based contactless exercise participation via an online platform during postpartum period on major metabolic hormone. (A-D) Bar graphs depict each value of insulin, leptin, cortisol, and serotonin before and after contactless exercise in non-exercised and exercised postpartum women. Two-way ANOVA followed by Tukey’s multiple comparisons test was used to compute adjusted p-values. Data are presented as mean ± SD and each circle represents an individual subject data. ***p < 0.001 versus corresponding pre-test; ###*p* < 0.001, ##*p* < 0.01 versus corresponding Ctrl (Non-exercise).

Previous studies have shown that postpartum women experience significant hormonal changes that may contribute to metabolic dysfunction, postpartum depression, or other health issues. Therefore, we measured the hormonal axes that may contribute to metabolic dysfunction and mood disorders in postpartum women (Fig. 1). A significant group-by-time interaction was identified between insulin (*F*_(1,116)_ = 4.716, *p* = 0.032, two-way ANOVA followed by Bonferroni’s post hoc test; Fig. 1A) and cortisol (*F*_(1,116)_ = 26.37, *p* < 0.001, two-way ANOVA followed by Bonferroni’s post hoc test; Fig. 1A) levels. Postpartum women assigned to continuous contactless exercise participation showed a greater reduction in serum insulin and cortisol levels in comparison to the corresponding pre-value (insulin: *p* < 0.001; cortisol: *p* < 0.001, Bonferroni’s multiple comparison test) and control (insulin: *p* = 0.003; cortisol: *p* < 0.001, Bonferroni’s multiple comparison test), although both non-exercised and exercised groups showed a significant decrease in the hormones after delivery (insulin: *p* = 0.003; cortisol: *p* = 0.004, Bonferroni’s multiple comparison test). However, there were no significant differences in leptin and serotonin levels, with a tendency to decrease leptin and increase serotonin levels after continuous contactless exercise participation.

### Evaluation of functional state and psychological factors

**Figure 2:**
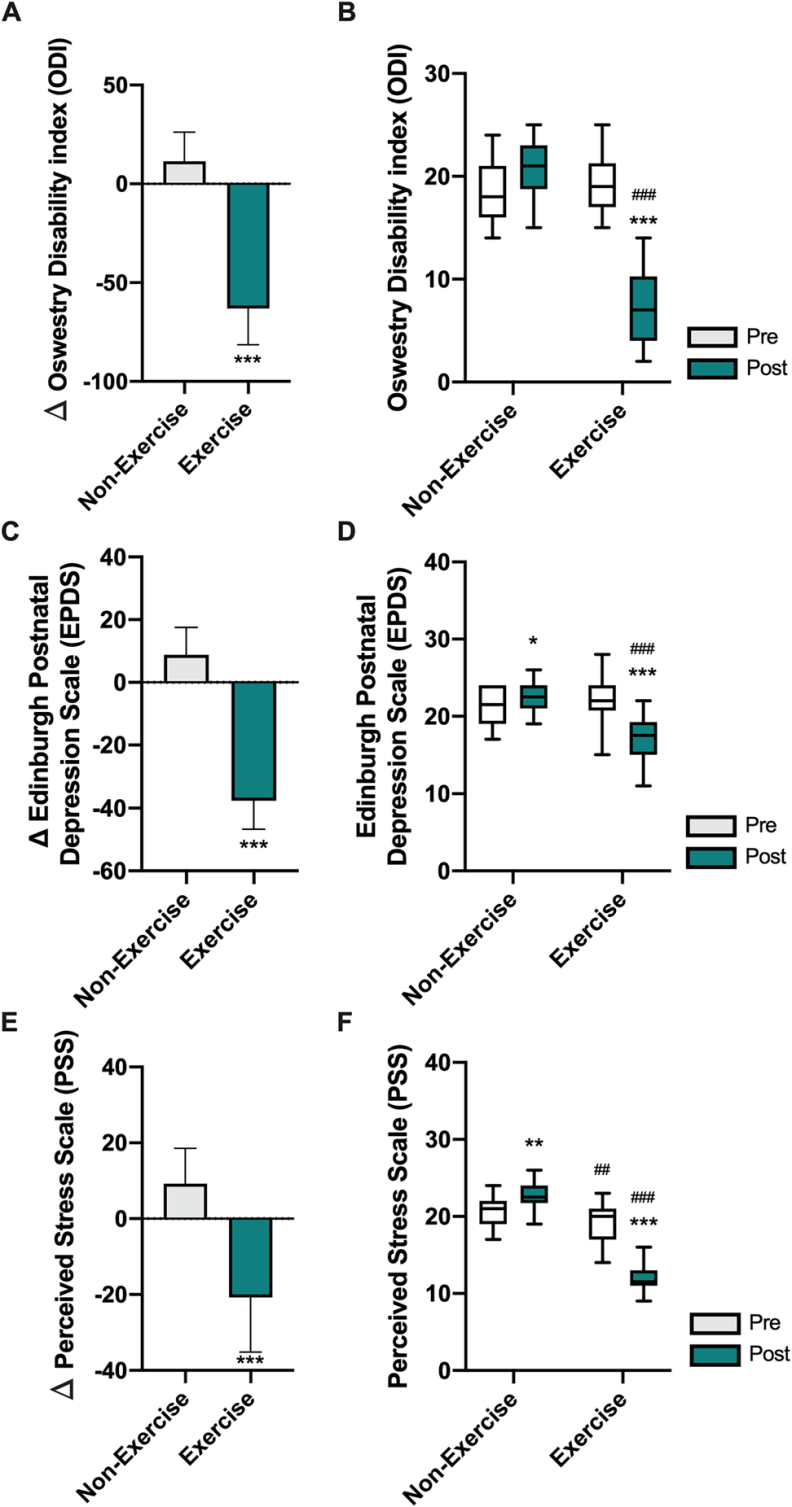
Self-report survey-based assessments results of stress and pain, and probability for depression in postpartum women. Bar graph depict delta (Δ) values showing the difference of post- to pre-contactless exercise of ODI (A), EPDS (C), and PSS (E) score. Unpaired t-test for ODI and PSS was used to calculate two-tailed p-values. Box plots depict median and interquartile range and whiskers show minimum and maximum values of ODI (B), EPDS (D), and PSS (F) score. Two-way ANOVA followed by Tukey’s multiple comparisons test was used to compute adjusted p-values. Data are presented as mean ± SD. ***p < 0.001, **p < 0.01, *p < 0.05 versus non-exercise group (unpaired t-test) or corresponding pre-test; ###*p* < 0.001, ##*p* < 0.01 versus corresponding Ctrl (Non-exercise).

After being assigned to home-based exercise, self-reported survey-based assessments of stress, pain, and the probability of depression in postpartum women were analyzed (Fig. 2). A significant group-by-time interaction was identified for all the indices (ODI: *F*_(1,116)_ = 169.1, *p* < 0.001, two-way ANOVA followed by Bonferroni’s post hoc test; EPDS: *F*_(1,116)_ = 46.36, *p* < 0.001, two-way ANOVA followed by Bonferroni’s post hoc test; PSS: *F*_(1,116)_ = 152.6, *p* < 0.001, two-way ANOVA followed by Bonferroni’s post hoc test; Fig. 2A, C, and E). During the postpartum period, continuous contactless exercise participation via an online platform resulted in a significantly decreased scores in ODI (t = 17.3, *p* < 0.001, two-tailed Mann–Whitney t-test, Fig. 2A), EPDS (t = 20.1, *p* < 0.001, two-tailed unpaired t-test, Fig. 2C), and PSS (t = 9.54, *p* < 0.001, two-tailed Mann–Whitney t-test, Fig. 2E), with a remarkable improvement in functional state (ODI: *F*_(1,116)_ = 169.1, *p* < 0.001, group-by-time interaction in two-way ANOVA followed by Bonferroni’s post hoc test, Fig. 2B) and emotional distress (EPDS: *F*_(1,116)_ = 46.36, *p* < 0.001, group-by-time interaction in two-way ANOVA followed by Bonferroni’s post hoc test; ΔPSS: *F*_(1,116)_ = 152.6, *p* < 0.001, group-by-time interaction in two-way ANOVA followed by Bonferroni’s post hoc test, Fig. 2D and F) in postpartum women.

### Association between biological parameters and psychological functioning

**Figure 3:**
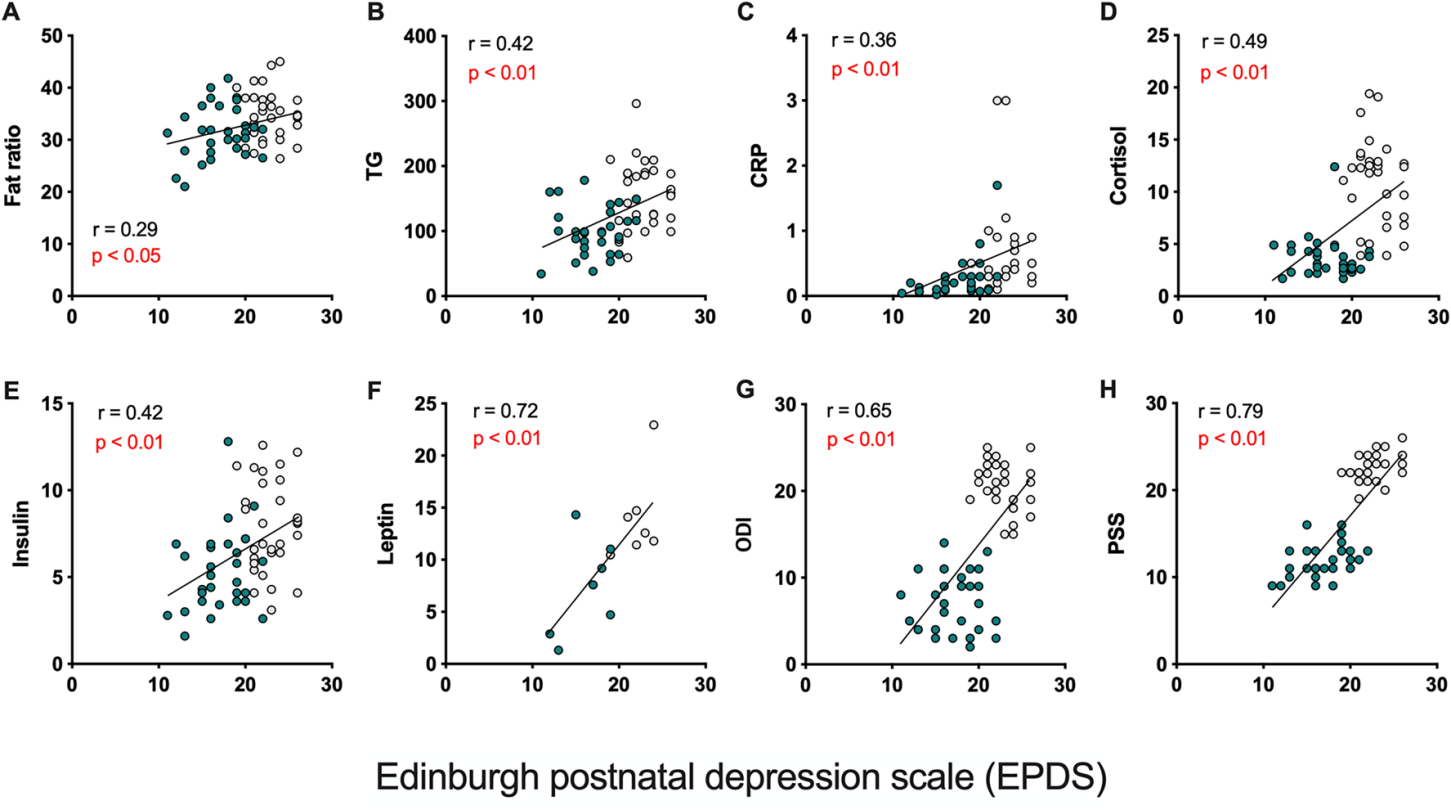
Association between biological parameters and postpartum depression. (A-H) Pearson’s correlation analysis was used to assess the association of biological parameters and EPDS score in non-exercised and exercised postpartum women. Two-tailed *p*- and *r*-values are depicted and the line in scatter plot shows a correlation between two variables. Each circle represents an individual subject.

Pearson’s correlation coefficient analysis was performed to determine whether changes in biological parameters mediated by continuous home-based contactless exercise participation via an online platform during the postpartum period were associated with emotional stress (EPDS and PSS) (Fig. 3). The EPDS scores were positively correlated with the fat ratio (r(58) = 0.15, *p* < 0.05), TG (r(58) = 0.42, *p* < 0.01), CRP (r(58) = 0.36, *p* < 0.01), cortisol (r(58) = 0.49, *p* < 0.01), insulin (r(58) = 0.42, *p* < 0.01), and leptin (r(12) = 0.72, *p* < 0.01), respectively as shown in Fig. 3A. Moreover, the PSS score was positively correlated with the fat ratio (r(58) = 0.33, *p* < 0.01), TG (r(58) = 0.56, *p* < 0.05), CRP (r(58) = 0.3, *p* < 0.05), cortisol (r(58) = 0.66, *p* < 0.01), insulin (r(58) = 0.39, *p* < 0.01), and leptin (r(12) = 0.67, *p* < 0.01), respectively and negatively correlated with HDL (r(58) =  − 0.25, *p* < 0.05), as shown in Supplementary Fig. S1. More importantly, EPDS and SPP scores were mutually correlated (see Supplementary Fig. S1 online).

## Discussion

Through a continuous excellence partitioning via an online platform (CEOP) study, we identified several notable results. First, exercise conducted during pregnancy and after childbirth alleviated postpartum depression by improving the pain and stress factors caused by the mother's physical structural imbalance. Second, the interactive contactless exercise lowered metabolic control and stress response factors (TG, Insulin, Leptin, and Cortisol) that contribute to postpartum depression. These results suggest that in a situation where social isolation caused by the COVID-19 pandemic is common, the interactive contactless exercise can alleviate postpartum depression by improving the metabolic imbalances that occur frequently after childbirth.

The COVID-19 pandemic has significantly impacted healthcare and daily life. In this intricate background, pregnant and postpartum women inevitably encountered distinctive challenges and adaptation. The pandemic’s disruptive effects extended to limited access to healthcare, altered support system, increased stress levels, and potential disruptions to routine prenatal and postnatal care. Expectants faced altered support networks as a result of social isolation, reshaping the communal connections integral to the fabric of pregnancy and postpartum recovery^33^. The uncertainties surrounding the pandemic, coupled with the inherent anxiety of pregnancy and childbirth, have potentially contributed to exacerbated feeling of isolation and stress levels. Within this dynamic context, exercise routines underwent transformations, responding to pandemic-related restrictions. These adaptations added layers of complexity to the landscape of physical activity during pregnancy and postpartum recovery^34^.

Regarding gestational weight gain (GWG) and obesity, the U.S. Centers for Disease Control and Prevention (CDC) and the World Health Organization (WHO) actively recommend participation in physical activities to maintain a healthy pregnancy; they also recommend at least 150 min of medium-intensity exercise per week for both pregnant and postpartum women^35,36^. To date, there are no clear guidelines for pregnant women's exercise; however, ACOG suggests that in healthy pregnant women, participating in moderate-intensity exercise for 20–30 min daily lowers insulin resistance and is effective in preventing gestational weight gain^21^. Nevertheless, there is a significant lack of alternatives for treating the obesity problem that was increased by the pandemic among pregnant women. Although there are many applications for home training methods and YouTube content, there are few real-time online exercise programs that consider the physical and epidemiological conditions of pregnant women, most of whom are healthy. Therefore, based on the exercise intensity of ACOG, we conducted a real-time (interactive) contactless exercise intervention for pregnant women under the supervision of a professional exercise leader and confirmed the following effects: The body fat amount, weight, BMI, and total cholesterol (TC) of this exercise group reduced more than those of the non-exercise group, and significant differences and interaction effects between groups and periods were observed for insulin and TG. These results are consistent with studies that show that online exercise reduces weight and blood lipids in overweight and obese pregnant women, lowering the risk of pregnancy complications^37,37^, pregnant women's real-time online Pilates participation during COVID-19 reduced their weight, BMI, and visceral fat after childbirth^38^. In addition, considering that the metabolic syndrome caused by obesity is caused by inflammation secreted by fat tissues, the increase in HDL, LDL, and CRP in this study indicates that exercise lowers the chain reaction of body fat-abnormal lipidemia-inflammation. In addition, significant differences in muscle strength between groups suggest that continuous exercise has a positive effect on the rate of postpartum recovery^39^, and previous studies support the idea that eHealth interventions have the same effect as face-to-face exercise^40,41^.

Psychological factors in pregnant and postpartum women are highly correlated with postpartum depression, and the COVID-19 environment has a higher risk of developing depression in young or pregnant women than in men^42,43^. Kim & Hyun suggested that online exercise is helpful for the psychological stability of motherhood^28^. However, to date, most studies on online exercises have been conducted on women after childbirth^44–46^. Therefore, we mediated continuous exercise during pregnancy and the postpartum period and verified its preventive effect on postpartum depression. The results showed that continuity exercise had intergroup differences and interaction effects on cortisol levels, PSS, and EPDS in women after childbirth, all of which were positively correlated with PPD. This indicates that continuous exercise in this study not only directly lowers EPDS but is also related to the subvariables of postpartum depression, including cortisol, PSS, and ODI. Previous studies have suggested that increased maternal stress has a significant impact on fetal neural development and cortisol levels, a potential biomarker of stress, are associated with exercise and cognitive development at six months of age^47^. Our findings confirm that exercise has a positive effect in cortisol levels, reflecting psychological stress and that cortisol reduction has a direct effect on postpartum depression. These results are consistent with studies showing that Maryam exercise has a positive effect on stress and serum cortisol levels in pregnant women and that the degree of activity (exercise frequency) in pregnant women has a negative correlation with cortisol levels^48,49^. In addition, in relation to hormones, a sub-variant of postpartum depression, leptin levels tended to decrease in the continuous exercise group of this study. These results are consistent with studies showing that exercise and dietary interventions in obese pregnant women significantly reduce leptin and that long-term exercise before and during pregnancy in animal experiments has a significant difference in insulin and leptin levels^50,51^. Bad habits, such as irregular postpartum sleep, late-night snacks, and binge eating, intensify depression by increasing leptin resistance and reducing serotonin^52^. Although there was no significant change in serotonin levels in this study, positive changes in leptin and cortisol levels indicated that continuous contactless exercise via an online platform can regulate the metabolic mechanism of postpartum depression.

Back pain is caused by physical and mechanical changes in women before and after pregnancy and has a direct effect on postpartum depression^53^. Although back pain is a common experience for most pregnant women, spinal rearrangement may not occur within first four to five months after childbirth. The severity of pain might either persist or worsen due to factors such as incorrect posture and habits, holding the baby on one side, or the overuse of smart devices^20,54^. According to a recent survey, 90% of early postpartum women (from postpartum to 21 weeks of age) who do not exercise experience abdominal muscle relaxation, which causes back pain, urinary incontinence, and pelvic girdle pain^55^. On the other hand, pregnant women who participate in regular physical activities relieve physical fatigue, back pain, and pelvic pain and reduce the risk of sleep disorders and depression^56,57^. In this study, back pain was positively correlated with postpartum depression, and continuous exercise directly reduced back pain. Considering that previous studies have suggested that back pain can promote pregnancy and postpartum depression^58^, home-based online training can be used as an indirect healing method. It will also be effective in reducing postpartum depression during continuous rather than short-term exercise. Although this study did not compare short-term and long-term interventions, this aspect should be verified through further studies.

Taken together, our research supports previous studies showing that online exercise is a good alternative for maintaining healthy conditions in pregnant and postpartum women^37,59^. It is also consistent with studies showing that online exercise programs are more effective when conducted under the supervision of leaders than when exercising alone^60,61^. In particular, the presence of a leader during exercising in pregnancy may exert a synergistic effect, enhancing the exercise effect through interactive communication and the establishment of consensus among the participants who share similar pregnancy and childbirth periods, as well as parenting stress. However, pregnant women with significant metabolic changes are vulnerable to various diseases; therefore, continuous monitoring and measures are needed throughout the pregnancy and postpartum period. In addition, it’s worth noting that certain studies have suggested that exercise may not confer specific advantages for the health of pregnant women and their offspring^62,63^. These conflicting results could be attributed to the unclear definition of exercise type or intensity in pregnant women, and dietary recommendations might hold significance than exercise concerning gestational weight gain^39,57,63^. Particularly in recent study, the discrepancies between the guidelines of the American College of Obstetricians and Gynecologists and the American College of Sports Medicine (ACSM) have been reported^64^. It has been highlighted that the guidance on determining optimal exercise intensity is extensive, potentially leading to confusion among pregnant individuals. Therefore, it is necessary to validate a diverse and integrated intervention through a more systematic classification according to pregnant women's weight gain, experience in exercise participation, pre-pregnancy, or quarterly group classification to refine and differentiate exercise guidelines for the direction of future studies.

Despite the various effects observed in this study, it has several limitations. First, it was not possible to consider individual differences in the physical strength levels of pregnant women. Since pre-pregnancy fitness levels can affect the gestational weight gain and postpartum depression and are also related to the rate of recovery after childbirth, exercise interventions considering pre-pregnancy BMI and fitness levels should be applied in future studies. However, as mentioned earlier, there are no detailed guidelines for online exercises for pregnant women. Therefore, it will be our homework to prepare exercise guidelines for non-face-to-face programs and verify their safety and effectiveness. Second, the dietary recommendations for pregnant and postpartum women were not thoroughly regulated. This study was conducted in a special environment of COVID-19, and the stress experienced by the participants greatly increased. Therefore, excessive calorie restriction can harm the psychological state of pregnant women, and increased stress is likely to cause participants to drop out of the study. We provided information on dietary recommendations on the day of the pre-examination, and after the study began, nutritional status was checked once a week by a professional nutritionist. The participants wrote a daily meal diary, and the nutritionist advised them about incorrect eating habits or food choices. However, some postpartum women's late-night snacks, high-glycemic index (GI) foods, and salty foods are not completely regulated. In future studies, balanced diet intervention and exercise should be combined to verify the preventive effects of postpartum depression. Third, the number of blood samples used in the analysis of some of the hormonal indicators (Leptin, Serotonin) was insufficient. This was the result of the subject's disapproval of the process of collecting additional blood, and the results of the experiment are planned to supplement the reliability of the results in future follow-up studies on the premise of a high possibility. Lastly, we assessed body composition using bioelectrical impedance analysis (BIA), In-Body 770 equipment, which offers a time- (within 2 min) and cost-effective, and high reproducibility (coefficient of variation, < 5%)^65–68^. Unfortunately, however, it is important to note that In-Body 770, despite its advantages, has limitations as suggested by Brewer et al.^69^. This limitation should be considered when interpreting our results, and future studies may explore alternative methods or combination approaches for a more comprehensive assessment.

In conclusion, these findings suggest that the contactless online exercise interventions can mitigate postpartum depression by addressing metabolic dysregulation that frequently occurs after delivery, especially in situations of social isolation caused by the pandemic.

## Methods

### Ethics approval

This study was approved by the Bioethics Committee of Korea National Sports University (IRB No. 1263-202109-br-018-01) and was conducted following the related guidelines and regulations (the recommendations of the Declaration of Helsinki). All participants were provided with a detailed description of the study, and they signed a consent form before the study began. Written informed consent was obtained from all subjects.

### Study design and participants

**Figure 4:**
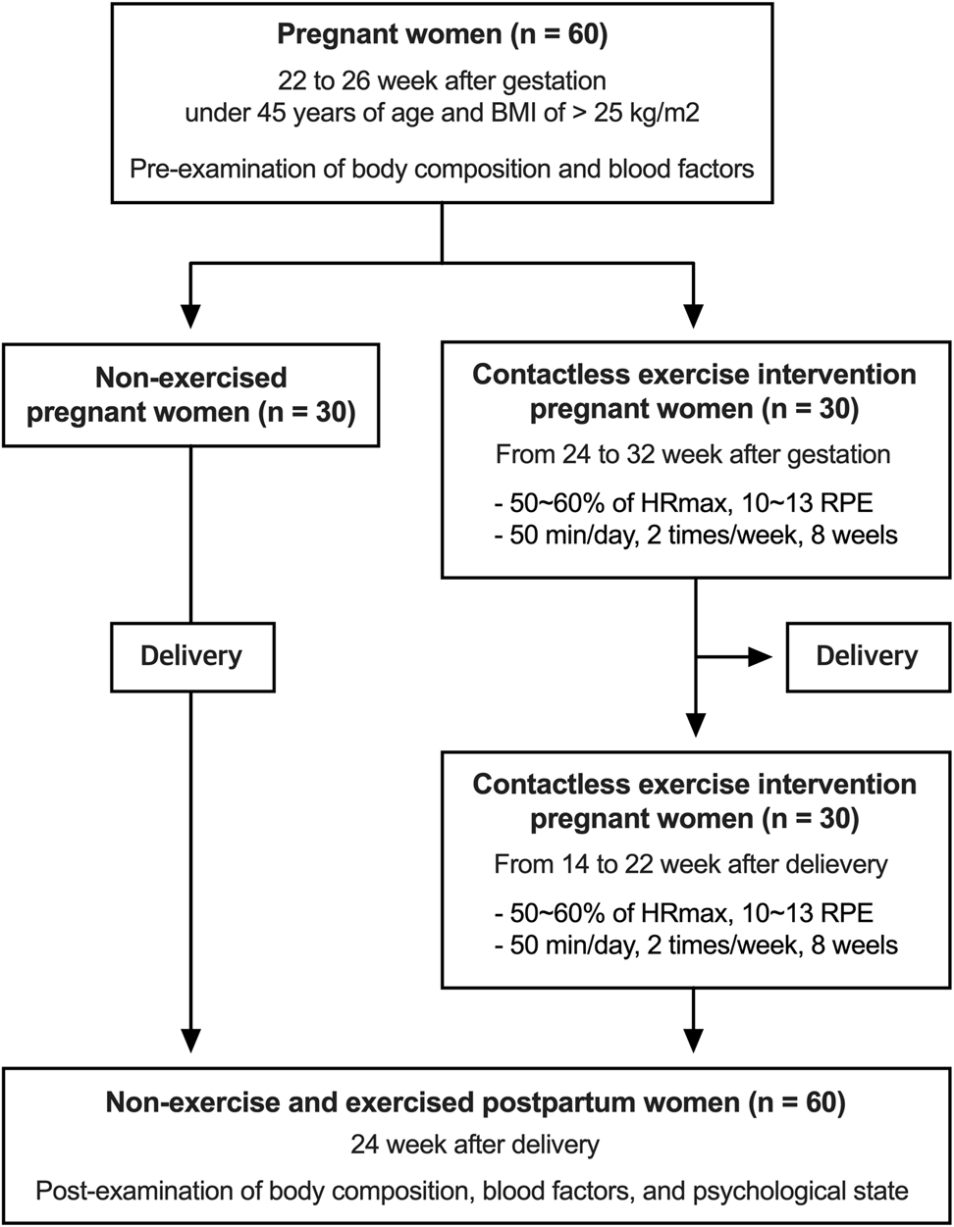
Flowchart of recruitment of eligible subject and contactless exercise intervention.

A total of 60 pregnant women at 22–26 weeks of pregnancy were included in the study, corresponding to those under 45 years of age and with a BMI of 25 kg/m^2^. In accordance with the guidelines from the American Obstetrics and Gynecology Association, which underscore the importance of tailoring exercise prescriptions to accommodate physical fitness of pregnant women, particularly those with overweight and obesity^24^, it is imperative to recognize that different weight categories necessitate distinct exercise guidelines, make BMI a pertinent inclusion in this study. All participants took part in the study with the consent of their doctors and had no medical findings (not corresponding to gestational diabetes or high blood pressure, excluding twin pregnant women). Since this study aimed to verify the effectiveness of continuous exercise during pregnancy and after childbirth, participants who could participate for a total of eight months were selected. This duration encapsulates distinct phases, including the exercise period during pregnancy, the postpartum recovery phase (extending from immediately after childbirth to 100 days postpartum, during which exercise is contraindicated), and the subsequent postpartum exercise phase, facilitating a comprehensive exploration of the impact of continuous exercise across the critical stages. A total of 60 pregnant women were recruited, and they were randomly divided into exercise (n = 30) and non-exercise groups (n = 30). Thirty pregnant women in the exercise group participated in Pilates for eight weeks, and after childbirth, they were recruited again and participated in postpartum Pilates for eight weeks. The Study design is illustrated in  Fig. 4 .

### Contactless exercise program

The exercise group engaged in a contactless exercise program consisting of Pilates over a 16-week period, with 8 weeks during pregnancy (first intervention) and 8 weeks re-starting 100 days after childbirth (second intervention). The participants participated in the exercise in real-time using a video chat application in their respective homes and communicated directly with the leader. The exercise was conducted for a total of eight weeks, twice a week, for 50 min a day, and it was the same before and after the prenatal period. The exercise program consisted of warm-up, main, and cool-down exercises with a rest time of 10 s between sets. The exercise intensity was set at 55–65% of the maximum heart rate, considered as light intensity according to the American College of Sports Medicine^70^. This in aligns with the guideline of the American Obstetrics and Gynecology Association and related meta-analysis, which recognizes that pregnant women typically experience a decrease in anaerobic and aerobic exercise capacity due to the blunted heart rate response, coupled with a reduction in pulmonary reserve^24,71^. Unfortunately, however, monitoring heart rate during exercise in pregnant women can be challenging. Therefore, based on relevant suggestion in previous study, which suggest that the use of ratings of perceived exertion may be a more effective means to monitor exercise intensity during pregnancy than heart-rate parameters, the exercise intensity assessment substantially maintained a subjective exercise intensity index, utilizing the rate of perceived exertion (RPE) on Borg’s scale, at a level of 11–13^24^. This involved a progressive increase in exercise intensity every three weeks, guided by the principles of progressive overload. The progression incorporated an increase in the number of repetitions and the inclusion of strength-oriented Pilates movements using body weight and weight-bearing resistance movement with elastic band and balls^24^. Movements rated at RPE 11–12 levels are perceived as challenging but not overly difficult for pregnant individuals. Considering the adopted exercise program, which include strength-oriented Pilates movements, a shorter rest interval ranging from 10 to 20 s between sets was deemed appropriate.

Regarding the supervision of the training sessions, the training sessions were supervised on an hourly basis, with the supervisor assessing the participants’ pain levels and ensuring the correct execution of exercise program immediately after each session. In cases where participants struggled to comprehend the exercise movements, detailed explanations and exercise videos were provided separately. Additionally, every two weeks, the supervisor inquired about physical fatigue or discomfort, energy levels, mood state. The effectiveness of the exercise was assessed to incorporate individualized management, which involved monitoring weight, nutritional status, and addressing specific needs. The non-exercise group banned participation in all sports except housework and childcare and was periodically supervised during the intervention period. The online Pilates program used in this study is shown in Table 2.

**Table 2:** Contactless exercise intervention program.

Modes	Contents	Time (min)	Reps, set, and rest	RPE
	Prenatal exercise			
Warm-up	Breathing & stretching	10		10
Main Exercise	**Level 1: 1–3 week** Bridge, clam, Cat cow, One -leg circles, Side kick, Spine twist, Kneeing push-up, Squat **Level 2: 4–6 week** Half standing roll down, Leg side up, Saw, Donkey kick, Down dog, Half-lunges, Wide-lunges, Side steps **Level 3: 7–8 week** Squat with mini ball, Spine twist on the ball, Shoulder press with band, Lunge-twist, kneeing down dog plank, Breathing	30	12–15 reps × 3 set 10–20 s rest between sets	11–13
Cool-down	Deep breathing & Meditation	10		10
	**Postnatal exercise**			
Warm-up	Total body stretching	10		10
Main Exercise	**Level 1: 1–3 week** Roll down, Kneeing push-up, Crunch, Leg raise, Side leg circles, Squat with shoulder press **Level 2: 4–6 week** Lunges, Sidewalk, Hill squat, Animal walking, Side-crunch, Wide leg raise, push-up **Level 3: 7–8 week** Deep squat, Lunge twist, Side plank, Knee up run, Squat Jump, Oblique Crunch, Leg raise with mini ball, Saw with ball	30	12–15 reps × 3 set. 10–20 s rest between sets	11–14
Cool-down	Breathing and stretching	10		10

### Body composition and blood tests

All participants stopped eating food two hours before the measurement and emptied their bladders 10 min before the test. The height of each participant was measured using an automatic kidney meter (DS-103M; Dong Shan Jenix Co., Seoul, Korea). Body composition tests measured weight (kg), body fat (kg), BMI (kg/m^2^), skeletal muscle mass (kg), and body fat percentage (%) using bioelectrical impedance analysis (In-Body 770, Biospace Co., Seoul, Korea). All the participants removed their metal accessories, dressed lightly, and stood with their sole touching the foot and hand electrodes. For blood sugar and blood lipid tests, all participants fasted from 9 pm on the previous day to 10 am on the day of the test. The nurse took a 5 mL blood sample from the upper arm vein. After incubating at room temperature for 30 min, the blood sample was centrifuged (3000 rpm, 10 min) to separate the serum and immediately transferred to the Green Cross Research Institute. The analyzed items included insulin, TC, TG, LDL, HDL, CRP, insulin, cortisol, leptin, and serotonin.

### Edinburgh Postnatal Depression Scale test

A postpartum depression test was performed using the Edinburgh Postpartum Depression Scale (EPDS). The EPDS is a self-report scale developed as a tool for screening depression, and its validity for use during pre-pregnancy period was established by Cox et al.^72^. In this investigation, a culturally adapted version for Korean women, as refined by Lee & Kwon, was utilized^73^. A total of ten question items, and questions about depression, anxiety, and suicidal thoughts over the past week were asked. Each item was evaluated on a 4-point Likert scale ranging from 0 to 4, with a total score of 0 to 30 (A total score of 13 points or higher was considered as risk of depression). The reliability of the EPDS, assessed by Cox et al. and Lee & Kweon, was reported as Cronbach’s α = 0.92 and α = 0.85, respectively^72,73^. In the present study, the Cronbach’s α was determined to be 0.83, which was analyzed using SPSS version 22.0 (IBM, Armonk, NY, USA).

### Perceived Stress Scale test

The Korean version of Perceptual Stress Scale (KPSS), initially developed by Cohen and modified by Lee et al. in Korean, was used for the stress test^74,75^. The total number of items was 10, and each item used a 5-point scale (0 = never, 1 = rarely, 2 = occasionally, 3 = frequently, 4 = Very Often) to ask about the perceived stress over the past month. Negative questions were scored in reverse, with a total score of 40. The higher the score, the more stressful it was. The PSS reliability of this study (Cronbach’s alpha) was 0.83, which was analyzed using SPSS version 22.0 (IBM, Armonk, NY, USA).

### Oswestry Disability Index test

The ODI (Oswestry Disability Index) questionnaire was used to measure the degree of back pain. The ODI was developed to measure the level of back pain symptoms and the degree of pain-related dysfunction, with a total of ten evaluation items consisting of pain management, personal management, walking, standing, sitting, sleep, and social life. Each question is scored from 0 to 5, and the total score is calculated by summing the scores for each item. Then, the total score is divided by 50 and multiplied by 100 to obtain the percentage value. Values in each range indicate patients with minimal, moderate, severe, paralysis, and bed-bound disabilities (0–20%, 21–40%, 41–60%, 61–80%, and 81–100%), respectively. To assess the effectiveness in this study, the scores were recorded both before and after the experiment. The reliability (Cronbach’s alpha) of the ODI, assessed by Shim, was reported as Cronbach’s α = 0.88^76^. In the present study, the Cronbach’s α was determined to be 0.84, which was analyzed using SPSS version 22.0 (IBM, Armonk, NY, USA).

### Statistical analyses

All data were processed using GraphPad Prism software (GraphPad Prism Inc., CA, USA). For assessment of the normality, Shapiro–Wilk and Kolmogorov–Smirnov test were performed (Supplementary table 1). Statistical significance was determined by two-way analysis of variance (ANOVA) to test the effect of time, group, and their interaction on functional state, psychological factors, body composition, and hormones, followed by Bonferroni’s post hoc test for multiple comparisons, but chi-square (χ^2^) test was performed if equality of variance was not assumed. An Unpaired t-test was employed to analyze the delta (Δ) changes in the EPDS, but the non-parametric Mann–Whitney test was performed to compare the ODI and PSS test since the data were not assumed to be normally distributed. Correlation between scores were derived from the psychological state questionnaire and related biological factors. The effect sizes (Cohen’s d) between the pre- and post-test results were expressed as the mean change and the values are considered as small (0.2), medium (0.5), or large (0.8 or greater) effective size. Statistical values were presented as mean ± standard deviation (*SD*), and a *p* < 0.05 was considered statistically significant.

### Ethical declarations

This study was approved by the Bioethics Committee of Korea National Sports University (IRB No. 1263-202109-br-018-01) and was conducted following the related guidelines and regulations (the recommendations of the Declaration of Helsinki). All participants were provided with a detailed description of the study, and they signed a consent form before the study began.

### Supplementary Information

Supplementary Information.

## Data Availability

The datasets used and/or analyzed during the current study available from the corresponding author on reasonable request.
