# Supplementary material for: Contactless exercise intervention in prenatal and postnatal period during COVID-19 lowers the risk of postpartum depression
Source: Sci Rep. 2024 Apr 29;14:9780. doi: 10.1038/s41598-024-60658-7 (PMC11058814; doi:10.1038/s41598-024-60658-7)
Supplement: Supplementary file 1 — Supplementary Information. [file 41598_2024_60658_MOESM1_ESM.docx]

Supporting Information

Contactless exercise intervention in prenatal and postnatal period during COVID-19 lowers the risk of postpartum depression

Dong-Joo Hwang^1,2^, Joon-Yong Cho^1^, Ah-Hyun Hyun^1^*

^1^Exercise Biochemistry Laboratory, Korea National Sport University, Seoul, Korea

^2^Sport Science Institute, Korea National Sport University, Seoul, Korea.

Contact information: [knupe838@knsu.ac.kr](mailto:knupe838@knsu.ac.kr)

This file include:

Supplementary figure 1 (Supplementary F1)

Supplementary table 1 (Supplementary T1)


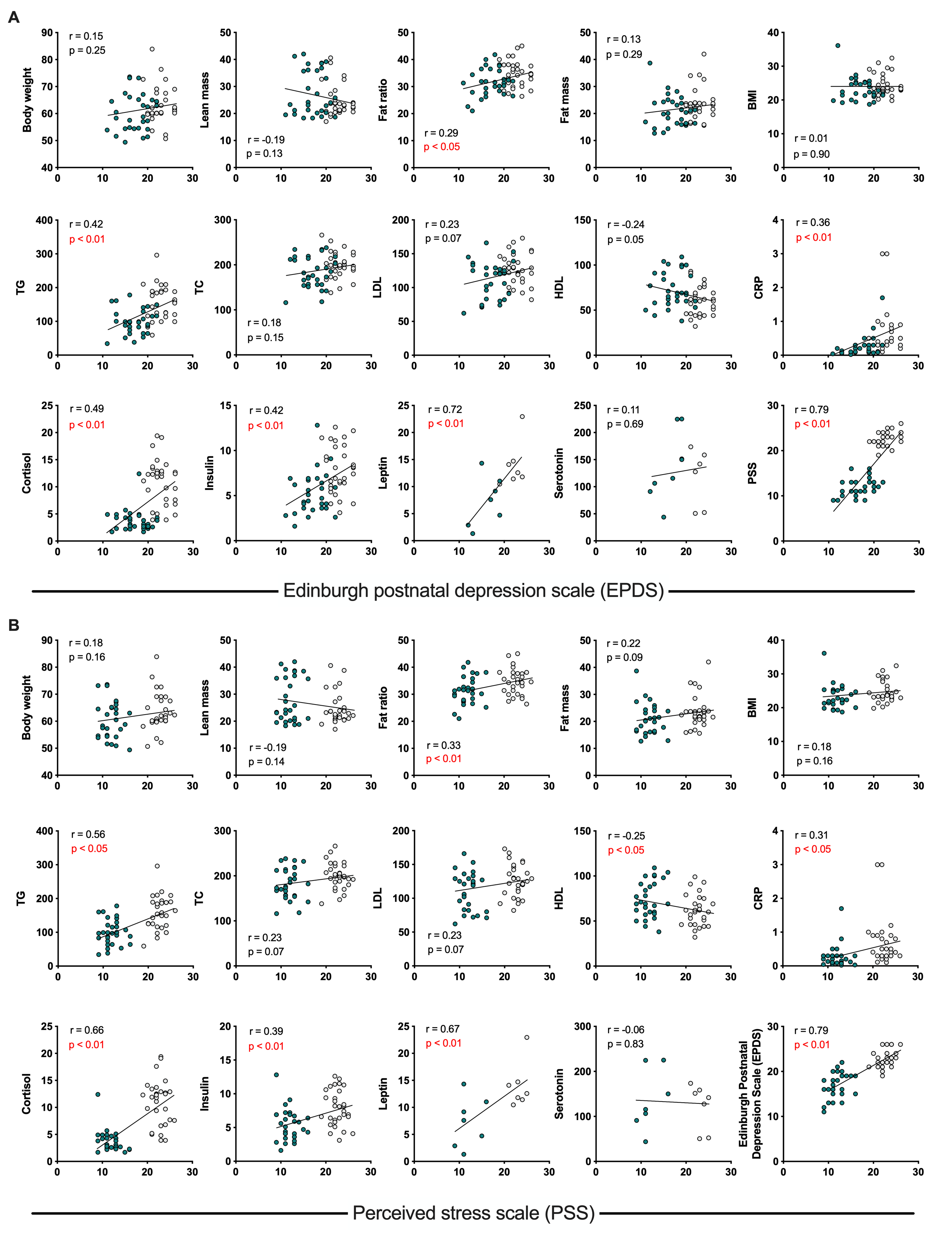


Supplementary Fig. S1. Association between metabolic parameters and perceived stress and postpartum depression. Pearson’s correlation analysis was used to assess the association of metabolic parameters and EPDS (A) and PSS (B) score in non-exercised and exercised postpartum women. Two-tailed *p*- and *r*-values are depicted and the line in scatter plot shows a correlation between two variables. Each circle represents an individual subject.

Supplementary table 1. Skewness, Kurtosis, and normality tests for variables

| **Variable** | **Skewness** | | |  | **Kurtosis** | | |  | ***P*** | |
| --- | --- | --- | --- | --- | --- | --- | --- | --- | --- | --- |
|  | **Value** | **SE** | **Z** |  | **Value** | **SE** | **Z** |  | **Kolmogorov-Smirnov test** | **Shapiro-wilk test** |
| Body weight | 0.399 | 0.221 | 1.805 |  | 0.150 | 0.438 | 0.343 |  | .530 | .134 |
| Fat mass | 1.209 | 0.221 | 5.472 |  | 2.664 | 0.438 | 6.077 |  | .000 | .000 |
| Lean mass | 0.955 | 0.221 | 4.325 |  | -0.471 | 0.438 | -1.074 |  | .000 | .000 |
| Fat ratio | -0.030 | 0.221 | -0.136 |  | -0.429 | 0.438 | -0.979 |  | .200 | .651 |
| BMI | 0.952 | 0.221 | 4.312 |  | 1.456 | 0.438 | 3.321 |  | .002 | .000 |
| TG | 0.314 | 0.221 | 1.423 |  | -0.484 | 0.438 | -1.105 |  | .060 | .088 |
| TC | 0.093 | 0.221 | 0.420 |  | -0.114 | 0.438 | -0.261 |  | .200 | .860 |
| HDL | 0.167 | 0.221 | 0.756 |  | -0.937 | 0.438 | -2.138 |  | .080 | .013 |
| LDL | -0.045 | 0.221 | -0.203 |  | -0.327 | 0.438 | -0.746 |  | .016 | .142 |
| CRP | 2.673 | 0.221 | 12.103 |  | 9.280 | 0.438 | 21.170 |  | .000 | .000 |
| Insulin | 0.269 | 0.221 | 1.219 |  | -0.468 | 0.438 | -1.068 |  | .007 | .034 |
| Cortisol | 0.039 | 0.221 | 0.178 |  | -1.172 | 0.438 | -2.674 |  | .001 | .000 |
| Serotonin | -0.124 | 0.427 | -0.291 |  | -0.697 | 0.833 | -0.837 |  | .161 | .290 |
| Leptin | 1.418 | 0.441 | 3.219 |  | 1.692 | 0.858 | 1.972 |  | .000 | .000 |
| ODI | -0.862 | 0.221 | -3.904 |  | -0.248 | 0.438 | -0.565 |  | .000 | .000 |
| EPDS | -0.103 | 0.221 | -0.466 |  | -0.493 | 0.438 | -1.124 |  | .022 | .072 |
| PSS | -0.672 | 0.221 | -3.041 |  | -0.781 | 0.438 | -1.781 |  | .000 | .000 |
| ODI (Δ) | -0.079 | 0.309 | -0.255 |  | -1.527 | 0.608 | -2.509 |  | .008 | .000 |
| EPDS (Δ) | -0.519 | 0.309 | -1.682 |  | -0.213 | 0.608 | -0.350 |  | .009 | .095 |
| PSS (Δ) | -0.018 | 0.309 | -0.058 |  | -1.489 | 0.608 | -2.446 |  | .000 | .000 |
| SE: Standard error, Z values: Critical ration of skewness and kurtosis, BMI: Body mass index, TG: Total glycerol, TC: Total cholesterol, HDL: High-density lipoprotein, LDL: Low-density lipoprotein, CRP: C-reactive protein, ODI: Oswestry disability index, EPDS: Edinburgh postnatal depression scale, PSS: Perceived stress scale. | | | | | | | | | | |
